# Elevation of body temperature during exercise enhances protection against dexamethasone‑induced skeletal muscle atrophy

**DOI:** 10.1113/EP093744

**Published:** 2026-08-28

**Authors:** Berkay Ozerklig, Senay Akin, Seda Olgaz Bingol, Bulent Okan Yildiz, Scott K. Powers, Ibrahim Turkel, Haydar A. Demirel

**Affiliations:** ^1^ Department of Exercise and Sport Sciences Exercise and Sport Physiology Division Faculty of Sport Sciences Hacettepe University Ankara Turkey; ^2^ Turkish Doping Control Center Hacettepe University Ankara Turkey; ^3^ Division of Endocrinology and Metabolism Hacettepe University School of Medicine Ankara Turkey; ^4^ Department of Applied Physiology and Kinesiology University of Florida Gainesville Florida USA; ^5^ Faculty of Sport Sciences Near East University Nicosia Cyprus

**Keywords:** body temperature, exercise, glucocorticoid, heat‐shock protein, muscle atrophy

## Abstract

Prolonged glucocorticoid exposure induces skeletal muscle atrophy through suppression of protein synthesis and activation of catabolic signalling pathways. Although exercise attenuates glucocorticoid‐induced muscle loss, whether exercise‐induced increases in body temperature contribute remains unclear. In this study, we examined whether exercise in different thermal conditions modulates skeletal muscle atrophy and intracellular signalling during glucocorticoid exposure. Female Sprague–Dawley rats (*n* = 48) were assigned to six groups: control (CON), dexamethasone‐treated (DEX), cold exercise (∼5°C; CE), cold exercise with dexamethasone (CED), warm exercise (25°C; WE) and warm exercise with dexamethasone. Exercise protocols were matched, and dexamethasone was administered for 5 days. Dexamethasone reduced plantaris muscle mass (by 17%, *P* < 0.0001) and fibre cross‐sectional area (27%, *P* < 0.0001). During dexamethasone treatment, exercise in a cold environment provided partial protection, with muscle mass higher than DEX (*P =* 0.0476), but both muscle mass and fibre CSA remained lower than CON (*P =* 0.0096 and *P =* 0.0215, respectively). In contrast, exercise in a warm environment preserved muscle mass and fibre CSA (no difference vs. CON) and resulted in higher muscle mass (*P =* 0.0070) and fibre CSA (*P* < 0.0001) than DEX. Exercise‐induced increases in rectal temperature were associated with higher Hsp72 and Hsp25 expression, partial preservation of Akt–FoxO3a signalling and reduced MuRF1 expression, whereas exercise in a cold environment showed minimal heat shock protein response and limited suppression of catabolic signalling. These findings indicate that exercise‐induced elevation of body temperature enhances protection against glucocorticoid‐induced skeletal muscle atrophy and support a role for heat‐associated cellular stress responses in modulating muscle protein turnover during glucocorticoid exposure.

## Abstract (highlights)

- **What is the central question of this study?**
  Does an exercise‐induced increase in body temperature contribute to protection against dexamethasone‐induced skeletal muscle atrophy, independent of exercise intensity?
- **What is the main finding and its importance?**
  Exercise attenuated dexamethasone‐induced skeletal muscle atrophy in both cold (∼5°C) and warm (25°C) conditions; however, exercise performed in a warm environment, which elicited a greater rise in body temperature, resulted in superior preservation of muscle mass, accompanied by increased expression of heat shock proteins (Hsp72 and Hsp25), partial preservation of Akt–FoxO3a signalling and attenuation of MuRF1 upregulation. These findings identify exercise‐induced elevation of body temperature as an important physiological modulator that augments the anti‐atrophic effects of exercise during glucocorticoid treatment.

## 1 INTRODUCTION

Glucocorticoids are frequently used to treat a variety of inflammatory conditions, such as rheumatoid arthritis, sepsis, chronic heart failure and chronic obstructive pulmonary disease. Nevertheless, prolonged usage of high doses is known to cause skeletal muscle atrophy. Evidence suggests that glucocorticoids suppress protein synthesis and activate protein degradation pathways in skeletal muscle (Braun & Marks, 2015; Hasselgren, 1999). Although several signalling pathways have been implicated, the mechanisms through which glucocorticoids promote skeletal muscle atrophy are not yet fully defined.

Skeletal muscle mass is regulated by a dynamic balance between protein synthesis and degradation, coordinated by multiple intracellular signalling pathways. Within this regulatory network, the IGF‐1–Akt–mTOR pathway plays a central role in controlling muscle protein turnover, and genetic or pharmacological activation of Akt has been shown to prevent skeletal muscle atrophy (Bodine et al., 2001). Beyond its anabolic effects mediated through mTOR, Akt suppresses proteolytic pathways by inhibiting FoxO transcription factors, thereby limiting the expression of atrogenes involved in the ubiquitin–proteasome system (Schiaffino & Mammucari, 2011). Accordingly, reduced Akt phosphorylation observed in various experimental atrophy models, including dexamethasone‐induced muscle atrophy, is associated with nuclear translocation of FoxO3a and subsequent activation of the ubiquitin–proteasome system, leading to enhanced proteolysis in skeletal muscle (Sandri et al., 2004; Senf et al., 2010).

Exercise is widely recognized as an effective intervention for preventing skeletal muscle atrophy and attenuating muscle loss, including in glucocorticoid‐induced atrophy models (Falduto et al., 1990; Kumar et al., 2009; Macedo et al., 2014). The protective effects of exercise are mediated through multiple complementary and partly overlapping mechanisms. These include modulation of key signalling pathways that regulate muscle protein synthesis and degradation (Kumar et al., 2009), enhancement of antioxidant defence systems to limit oxidative stress (Powers et al., 2020) and improvements in mitochondrial function and quality control processes (Hood et al., 2019).

In addition to these established mechanisms, exercise is inherently accompanied by an increase in body temperature, which might represent an additional physiological factor influencing muscle protein homeostasis. Evidence from cellular heat stress models indicates that elevated temperature can modulate translational regulation by altering ribosomal activity and translation dynamics (Shalgi et al., 2013). Furthermore, whole‐body heat stress applied in conjunction with resistance exercise has been reported to coincide with activation of Akt–mTOR signalling in human skeletal muscle (Fennel et al., 2023), suggesting a potential interaction between thermal stress and anabolic signalling pathways.

One of the most consistent cellular responses to both exercise and heat stress is the induction of heat shock proteins (Hsps), particularly the 72 kDa isoform, Hsp72 (Demirel et al., 2001). Acting as a molecular chaperone, Hsp72 supports protein stability, limits cellular stress responses and enhances cellular resilience. Experimental studies have shown that increased Hsp72 expression is associated with protection against various stressors, including ischaemia–reperfusion injury and glucocorticoid‐induced skeletal muscle atrophy, and influences atrophy‐related signalling pathways in skeletal muscle (Demirel et al., 2001; Morimoto et al., 2015; Naito et al., 2000; Smuder et al., 2019). In addition, overexpression of Hsp72 has been reported to preserve Akt phosphorylation during glucocorticoid exposure, thereby potentially limiting catabolic signalling (Kukreti et al., 2013).

Alongside Hsp72, small heat shock proteins, such as Hsp25, have also been implicated in cellular stress responses, particularly through roles in cytoskeletal stabilization and cellular stress tolerance.  Hsp72 is closely associated with heat‐induced stress responses, whereas Hsp25 might also be regulated by exercise‐associated changes in body temperature and cellular stress conditions, thereby providing complementary insight into heat shock protein responses in different thermal environments (Akin et al., 2017; Noble et al., 2008). Hsp25 has also been suggested to influence catabolic signalling pathways, including nuclear factor‐κB (NF‐κB)‐mediated Muscle RING‐finger protein‐1 (MuRF1) expression, indicating a potential role in modulating muscle protein degradation in stress conditions (Dodd et al., 2009).

Although exercise activates multiple protective pathways, it remains unclear to what extent exercise‐induced elevations in body temperature contribute to the attenuation of glucocorticoid‐induced skeletal muscle atrophy. To address this, in the present study we examined the effects of a short‐term exercise protocol performed in distinct thermal conditions that elicit different temperature responses. We evaluated whether these temperature‐dependent differences are associated with changes in Hsp72 and Hsp25 expression, in addition to key signalling pathways regulating muscle protein turnover. This approach was designed to isolate the contribution of temperature‐related mechanisms within the multifactorial physiological response to exercise.

## 2 MATERIALS AND METHODS

### 2.1 Ethical approval

All the animal experiments were conducted under the approval of the Animal Ethics Committee of Hacettepe University, following the guidelines of the National Institutes of Health for the care and use of laboratory animals (approval no: 2019/04‐05).

### 2.2 Animals

**FIGURE 1:**
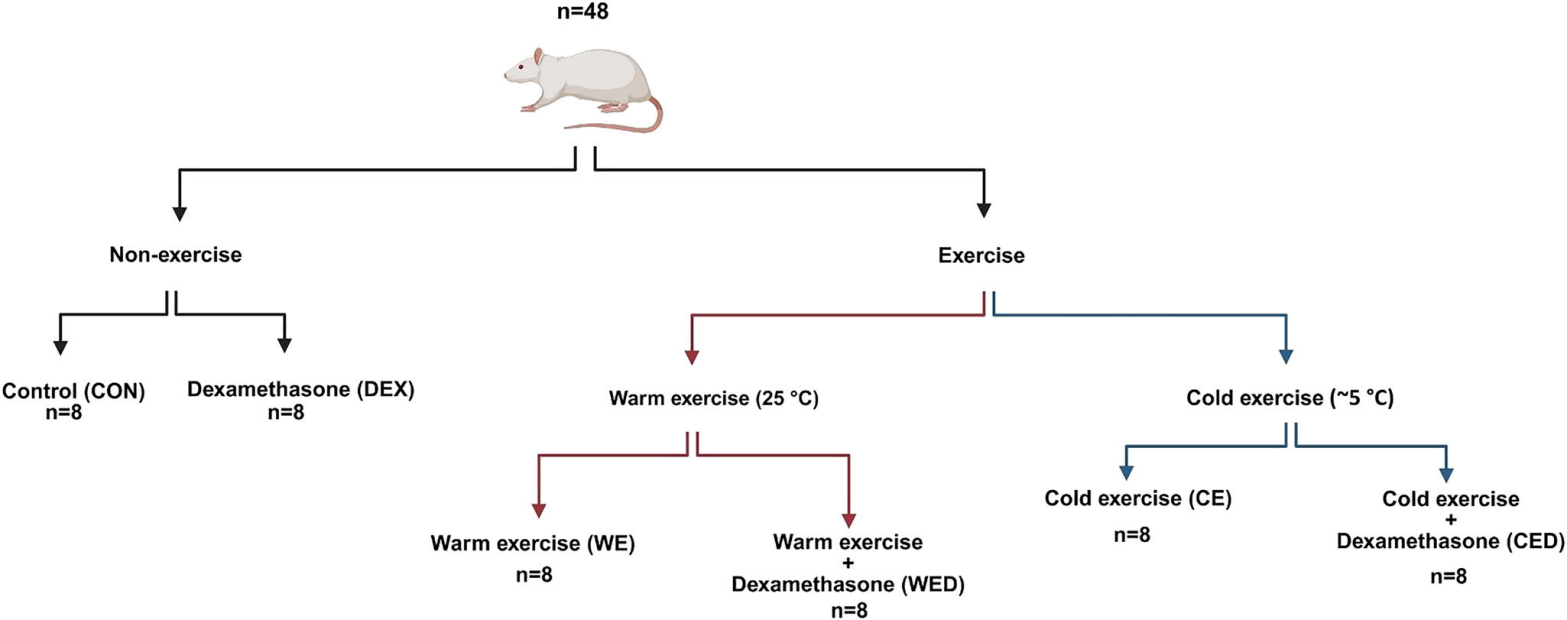
Experimental groups. Female Sprague–Dawley rats (*n* = 48) were randomly assigned to six groups (*n* = 8 per group): non‑exercise groups, including sedentary control (CON) and dexamethasone‑treated (DEX) rats; and exercise groups, including cold‑environment exercise (∼5°C; CE), cold‑environment exercise with dexamethasone treatment (CED), warm‑environment exercise (25°C; WE) and warm‑environment exercise with dexamethasone treatment (WED).

Female 3‐month‐old Sprague–Dawley rats were obtained from the Animal Facility of Gazi University. All animals were housed under a 12 h:12 h light–dark cycle at a controlled ambient temperature of 22°C ± 2°C and 50%–60 % relative humidity, with ad libitum access to standard chow (Carfil Rat & Mice Maintenance, Carfil Quality, Belgium; calories from protein, 20.67%; carbohydrate, 69.65%; and fat, 9.68%) and water. After a 1‐week acclimation period, rats were assigned randomly to the following groups: sedentary control (CON, *n* = 8); dexamethasone‐treated (DEX, *n* = 8); exercise performed at ∼5°C (CE, *n* = 8); exercise performed at ∼5°C with dexamethasone (CED, *n* = 8); exercise performed at 25°C (WE, *n* = 8); or exercise performed at 25°C with dexamethasone (WED, *n* = 8) (Figure 1). The CE and WE groups were included specifically to assess temperature‐dependent exercise responses (e.g., Hsp72, alongside Hsp25 expression), providing a basis for comparison with dexamethasone‐treated groups. All exercise protocols were conducted at the onset of the nocturnal (dark) cycle.

### 2.3 Dexamethasone treatment

Three groups (DEX, CED and WED; *n* = 8 per group) received daily subcutaneous injections of dexamethasone sodium phosphate (1 mg kg^−1^) for 5 days consecutively to induce skeletal muscle atrophy (Fappi et al., 2019; Ma et al., 2003). All other groups (CON, CE and WE) received subcutaneous injections of 0.9% saline in an equivalent volume.

### 2.4 Treadmill exercise protocol

**FIGURE 2:**
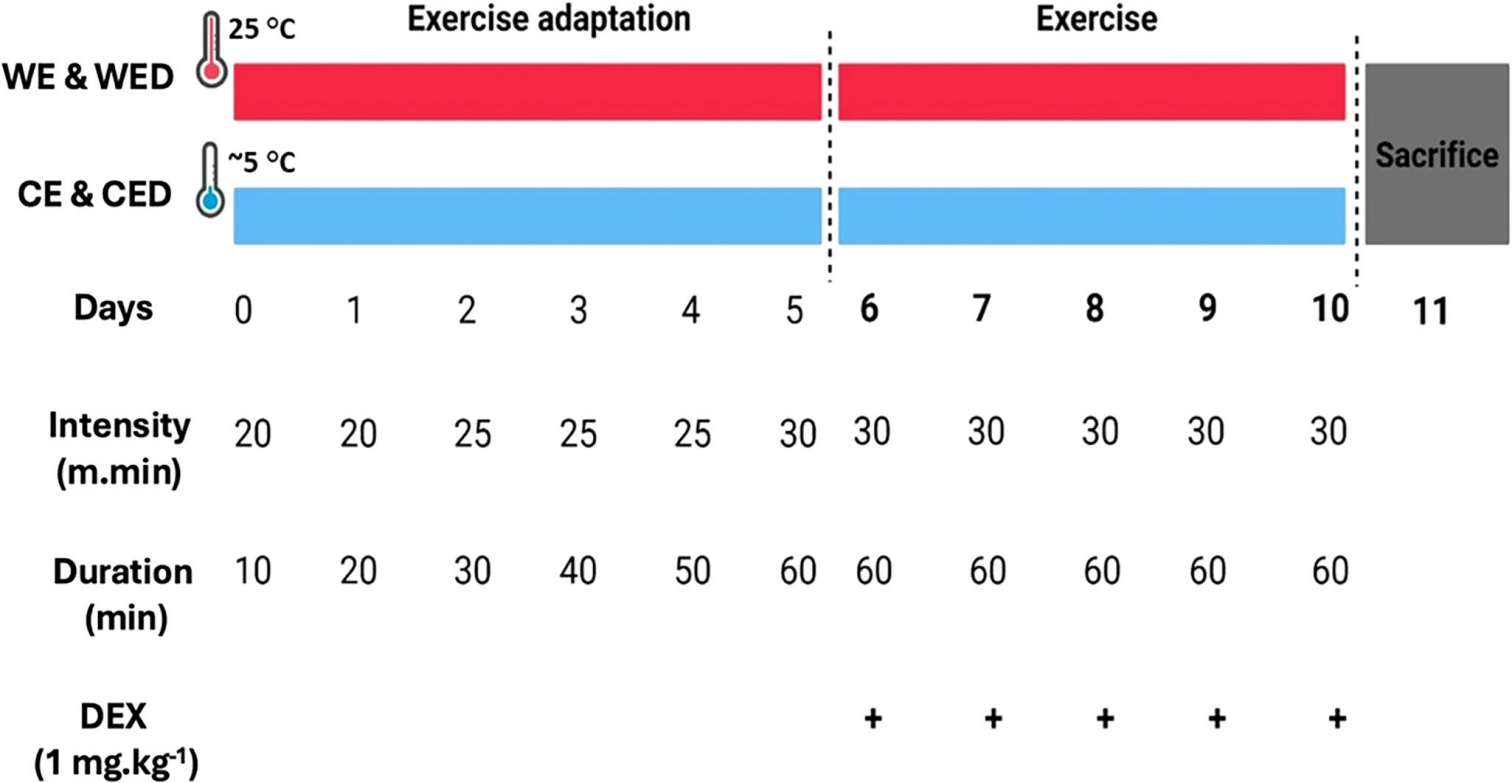
Experimental design and group allocation. Female Sprague–Dawley rats (*n* = 48) were randomly assigned to six groups (*n* = 8/group): sedentary control (CON), dexamethasone only (DEX), warm‑environment exercise (WE), warm‑environment exercise + dexamethasone (WED), cold‑environment exercise (CE) and cold‑environment exercise + dexamethasone (CED). All exercise groups first underwent a treadmill habituation period (days 0–5), during which running speed and duration were gradually increased. This was followed by a short‐term exercise phase (days 5–10), during which rats performed treadmill running at a constant intensity of 30 m min^−1^ for 60 min day^−1^. Exercise was performed at either 25°C (WE/WED) or ∼5°C (CE/CED). During the exercise phase, dexamethasone (1 mg kg^−1^) was administered daily by subcutaneous injection to the DEX, CED and WED groups. Animals were killed on day 11 (24 h after the final session).

Exercising animals were initially habituated to treadmill running at a speed of 20–25 m min^−1^ with no incline for 10 min. Exercise duration and intensity were increased progressively each day, reaching a final workload of 30 m min^−1^ for 60 min by day 5. Thereafter, rats performed 5 days consecutively of treadmill exercise at 30 m min^−1^ for 60 min day^−1^, corresponding to an estimated workload of ∼70% of maximal oxygen consumption (Criswell et al., 1993). During days 6–10, dexamethasone (1 mg kg^−1^) was administered once daily by subcutaneous injection to the DEX, CED and WED groups. All exercise sessions were performed on a motor‐driven treadmill in a climate‐controlled room, with the temperature at either 25°C or ∼5°C depending on group assignment. Ambient temperature was monitored continuously during exercise sessions. The cold condition averaged 5.1°C ± 0.8°C (range: 4.0°C–7.8°C), whereas the warm condition was maintained at 25°C with minimal variation. During the final 5 days of the exercise training protocol, rats in the CED and WED groups received daily subcutaneous dexamethasone injections (Figure 2).

The running speed and duration were identical for all exercise groups. Control and dexamethasone‐only animals were placed on a stationary treadmill for 10 min day^−1^ to control for handling stress. Rectal temperature was measured using a calibrated thermistor probe before and after each daily exercise session.

The exercise protocol used in this study was a minor modification of previously published protocols that have been shown to induce significant increases in Hsp72 expression in gastrocnemius muscle (Ogura et al., 2008). Animals were anaesthetized via intraperitoneal injection of ketamine/xylazine (90/10 mg kg^−1^ body weight) 24 h after completion of the exercise training. The depth of anaesthesia was confirmed before any invasive procedure by the absence of both pedal withdrawal and corneal reflexes. While the animals were maintained under deep general anaesthesia, the hearts were rapidly excised. The plantaris muscle was rapidly excised, cleared of tendons and visible fat, immediately frozen in liquid nitrogen, and stored at −80°C until further analysis.

### 2.5 Determination of corticosterone levels

Serum corticosterone levels were measured using ultraperformance liquid chromatography–tandem mass spectrometry with a Waters Acquity Triple Quadrupole system (Waters, Milford, MA, USA) equipped with an Acquity UPLC BEH C18 column. Mefruside was used as the internal standard. Corticosterone quantification was achieved by selective reaction monitoring of the precursor‑to‑product ion transition at *m*/*z* 347.2 → 329.2.

### 2.6 Tissue preparation

Plantaris muscle (∼50 mg) was homogenized 1:10 (wt/vol) in 100 mM HEPES (pH 7.9), 10 mM KCl, 0.1 mM EDTA (pH 8.0), 0.5 mM MgCl_2_, 1 mM dithiothreitol, 0.1 mM PMSF, 0.05% NP40, with a protease (Sigma‐Aldrich) and phosphatase inhibitor cocktail (Thermo Scientific) and centrifuged at 7500*g* for 15 min at 4°C. The resulting supernatant was collected. Plantaris protein content was assessed using the bicinchoninic acid (BCA, Takara Bio).

### 2.7 Immunoblot analyses

Protein extracts from the plantaris muscle (40 µg) were separated by SDS‐PAGE using 8%, 10% or 12% polyacrylamide gels for ∼2 h at 130 V. Following electrophoresis, proteins were transferred onto nitrocellulose membranes using a semi‐dry Trans‐Blot Turbo system (Bio‐Rad) for 30 min at 25 V. Membranes were blocked for 1 h at room temperature in Tris‐buffered saline containing 0.1% Tween‐20 and 5% non‐fat dry milk to prevent non‐specific binding. Subsequently, membranes were incubated overnight at 4°C with primary antibodies against Hsp25 (ADI‐SPA‐801, StressGen, 1:1000), Hsp72 (SPA‐810AP, StressGen, 1:1000), Akt (#9272, Cell Signaling Technology, 1:1000), phosphorylated Akt (Ser473; #9271, Cell Signaling Technology, 1:1000), FoxO3a (#2497, Cell Signaling Technology, 1:1000), phosphorylated FoxO3a (Ser253) (#9466, Cell Signaling Technology, 1:1000) and MuRF1 (#AF5366, R&D Systems, 1:1000). After incubation, membranes were washed thoroughly with Tris‐buffered saline containing 0.1% Tween‐20, then incubated with appropriate secondary antibodies (#7074, Cell Signaling Technology, 1:2000). Protein bands were visualized using a chemiluminescence detection system (ChemiDoc, Bio‐Rad). Band intensities were quantified by computerized densitometric analysis using ImageJ software (v.10.2; National Institutes of Health, Bethesda, MD, USA).

### 2.8 Measurement of plantaris myofibre cross‐sectional area

Serial cross‐sections (10 µm thick) of frozen plantaris muscle samples were obtained using a cryostat at −19°C, and standard Haematoxylin and Eosin staining was performed to assess myofibre cross‐sectional area (CSA), as previously described (Brooks & Faulkner, 1988). Sections were captured using a light microscope (ECLIPSE Ni series, Nikon). Myofibre CSA was determined using NIS‐Elements software by tracing the outlines of individual fibres. Mean fibre CSA was calculated by dividing the total fibre area by the total number of analysed fibres and expressed in micrometres squared (>300 muscle fibres analysed per muscle section).

### 2.9 Statistical analysis

Data are presented as the mean ± SD. Normality was assessed with the Shapiro–Wilk test, and no significant departures from normality were detected. Rectal temperature data were analysed using two‑way ANOVA. When a significant interaction was detected, simple‑effects analyses with Tukey's adjustment were performed, and significant main effects were examined further using Tukey's pairwise comparisons. All other outcomes were analysed with one‑way ANOVA, followed by Tukey's *post hoc* multiple comparisons test only when a significant main effect was observed. Individual data points are shown where appropriate. Analyses and figures were generated in GraphPad Prism (GraphPad Software, San Diego, CA, USA). Statistical significance was set at *P* < 0.05.

## 3 RESULTS

**FIGURE 3:**
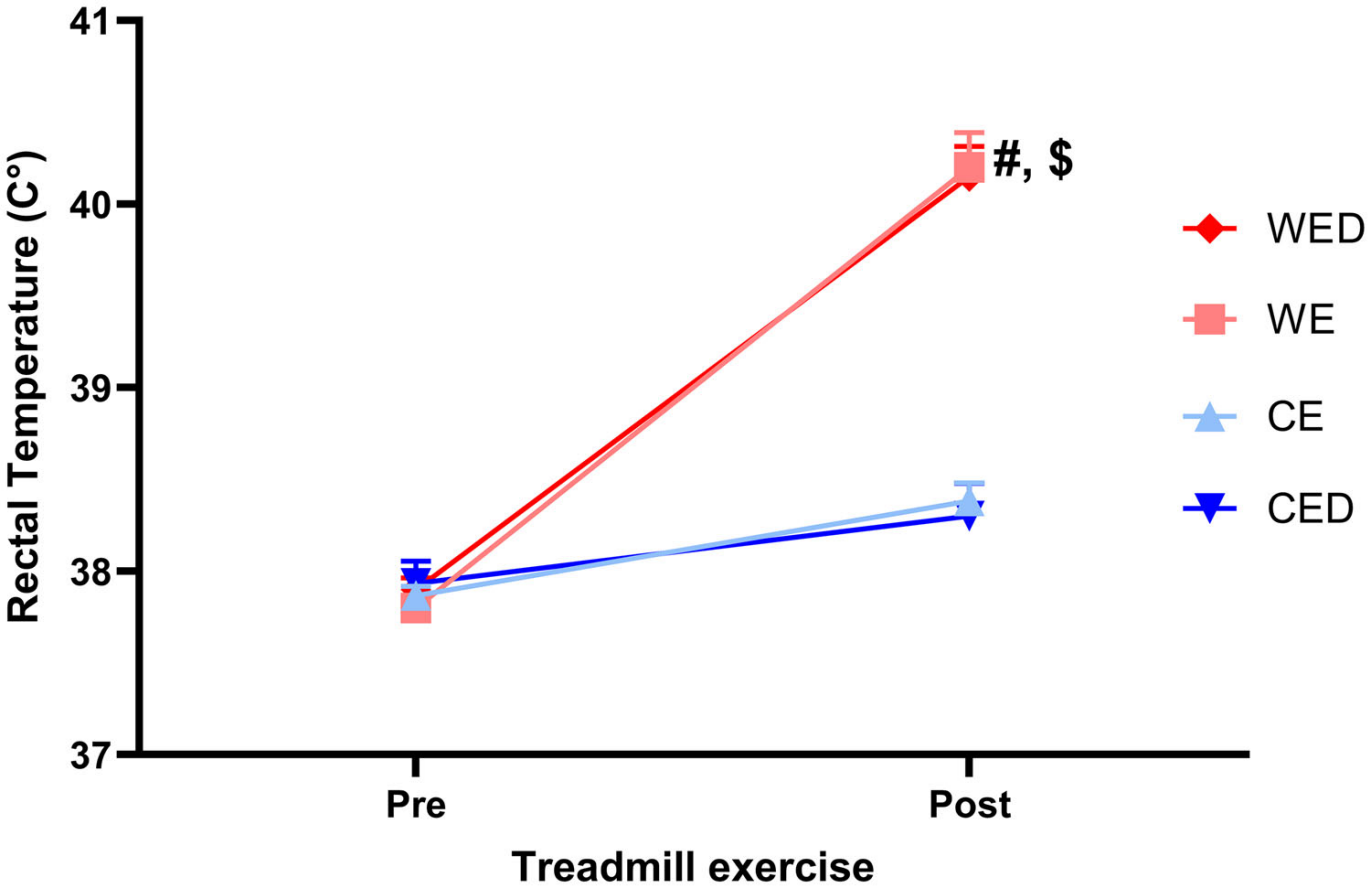
Changes in rectal temperature in exercising animals in cold (∼5°C) and warm (25°C) conditions. Rectal temperatures were recorded immediately before (Pre) and after (Post) the treadmill exercise protocol. Data are presented as the mean ± SD (*n* = 8 per group). Differences of the rectal temperature were analysed using two‐way ANOVA followed by Tukey's *post hoc* test. Symbols indicate statistical comparisons: # represents post vs. pre within the same group (*P* < 0.0001), and $ represents warm vs. cold group differences at the post time point (*P* < 0.0001). Abbreviations: CE, exercise in cold environmental conditions; CED, cold exercise with dexamethasone; CON, control; DEX, dexamethasone‐treated; WE, exercise in warm environmental conditions; WED, warm exercise with dexamethasone.

Pre‑ and postexercise rectal temperatures (mean ± SD) are shown in Figure 3. In the warm‐environment groups (25°C), rectal temperature increased from 37.8°C ± 0.1°C to 40.2°C ± 0.2°C in WE and from 37.9°C ± 0.1°C to 40.1°C ± 0.1°C in WED (both *P* < 0.0001). In the cold‑environment groups (∼5°C), rectal temperature changed from 37.9°C ± 0.1°C to 38.4°C ± 0.1°C in CE and from 37.9°C ± 0.1°C to 38.3°C ± 0.2°C in CED (*P =* 0.6720).

### 3.1 Exercise at a warm temperature attenuates glucocorticoid‐induced skeletal muscle atrophy

**FIGURE 4:**
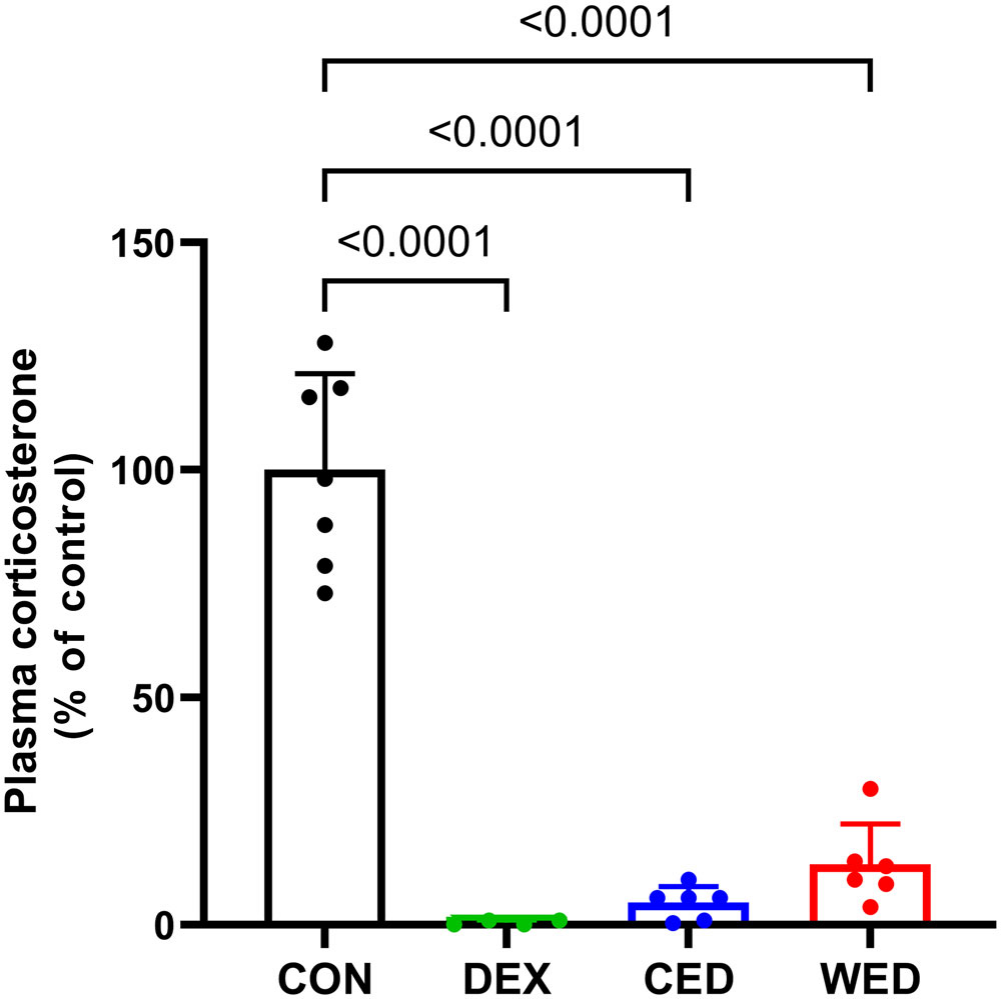
Plasma corticosterone levels. Corticosterone levels were significantly lower in all groups compared with the controls. Data are presented as individual data points (*n* = 4–7 per group). Differences were analysed using one‐way ANOVA followed by Tukey's *post hoc* test. Abbreviations: CE, exercise in cold environmental conditions; CED, cold exercise with dexamethasone; CON, control; DEX, dexamethasone‐treated; WE, exercise in warm environmental conditions; WED, warm exercise with dexamethasone.

As a validation of the glucocorticoid treatment, plasma corticosterone levels were measured before the assessment of skeletal muscle atrophy (Figure 4). Exogenous dexamethasone administration markedly suppressed endogenous corticosterone production compared with the control animals, confirming the effectiveness of the glucocorticoid treatment.

Skeletal muscle atrophy was evaluated using the ratio of plantaris muscle weight to body weight and myofibre CSA in dexamethasone‐treated groups (DEX, CED and WED).

**FIGURE 5:**
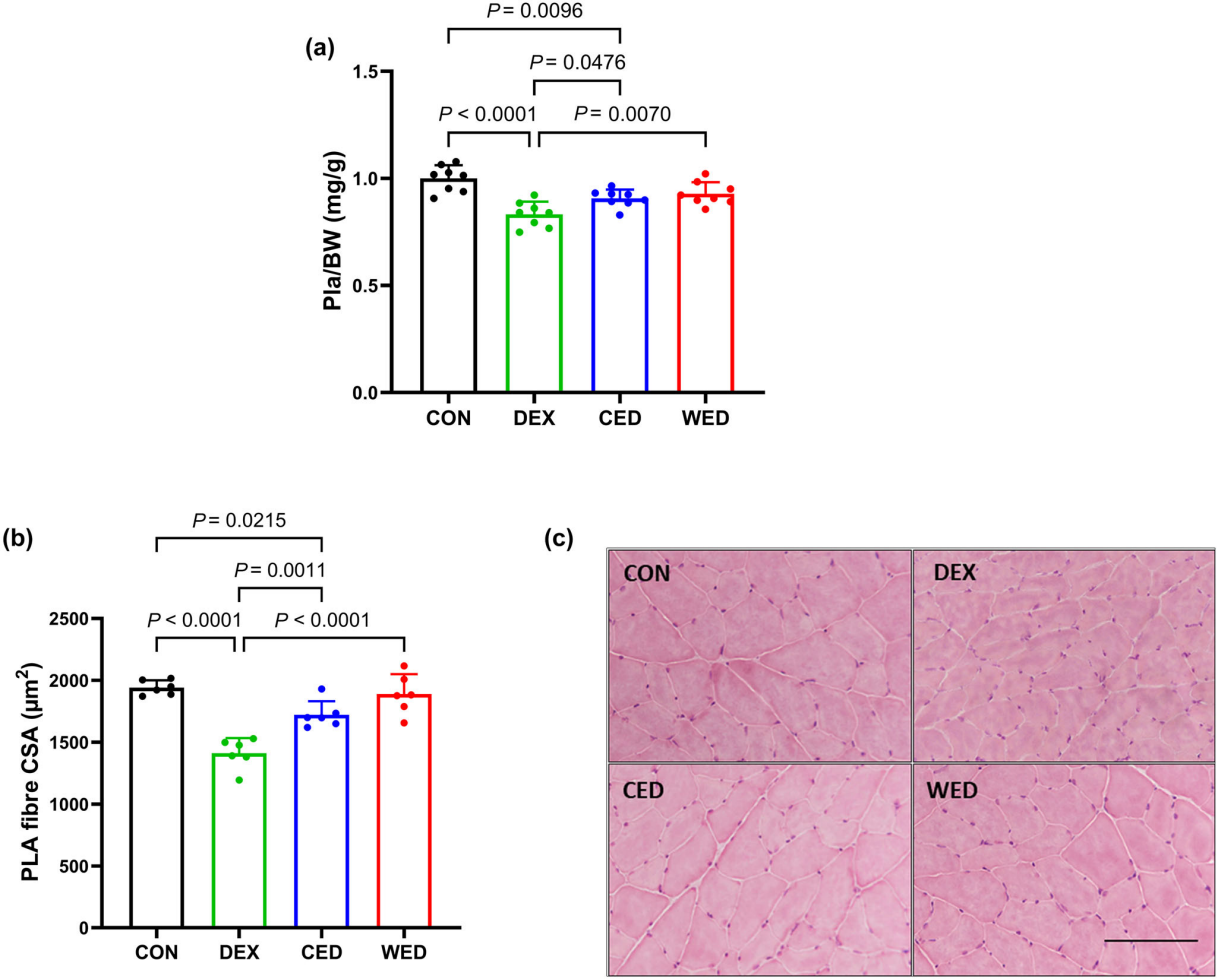
The effect of exercise‐induced body temperature on dexamethasone‐induced muscle mass reduction: (a) plantaris muscle weight‐to‐body weight ratio; (b) plantaris muscle fibre CSA; and (c) representative Haematoxylin‑ and Eosin‐stained cross‑sections of the plantaris muscle from the CON, DEX, CED and WED groups (*n* = 6–8). Abbreviations: BW, body weight; CE, exercise in cold environmental conditions; CED, cold exercise with dexamethasone; CON, control; CSA, cross‐sectional area; DEX, dexamethasone‐treated; PLA, plantaris muscle; WE, exercise in warm environmental conditions; WED, warm exercise with dexamethasone.

As shown in Figure 5a, the plantaris muscle weight ratio was significantly reduced in the dexamethasone group compared with controls (CON vs. DEX, *P* < 0.0001), corresponding to ∼17% decrease. A reduction was also observed in the cold exercise plus dexamethasone group (CON vs. CED, *P =* 0.0096), with a modest reduction (∼9%), whereas the warm‐environment exercise plus dexamethasone group (WED) did not differ from control, despite a small numerical reduction (∼7%). Both exercise groups exhibited higher muscle weight ratios than the dexamethasone‐only group, with a more pronounced effect in the 25°C conditions (CED vs. DEX, *P =* 0.0476; WED vs. DEX, *P =* 0.0070).

Likewise, as shown in Figure 5b, the CSA of muscle fibres was significantly reduced in the dexamethasone group compared with controls (CON vs. DEX, *P* < 0.0001), representing ∼ 27% decrease. The reduction was less pronounced in the CED group (CON vs. CED, *P =* 0.0215), with a decrease of ∼11%, whereas no significant reduction was observed in the WED group, despite a minor numerical decrease (∼3%), indicating preservation of fibre CSA (Figure 5b). Representative histological images are presented in Figure 5c.

Collectively, these results indicate that exercise attenuates dexamethasone‐induced skeletal muscle atrophy in both temperature conditions, with muscle mass and myofibre size remaining significantly reduced in the CED compared with CON, whereas muscle mass and myofibre size values in the warm environment exercise group did not differ significantly from controls.

### 3.2 Elevation of body temperature is associated with Hsp induction in plantaris muscle

**FIGURE 6:**
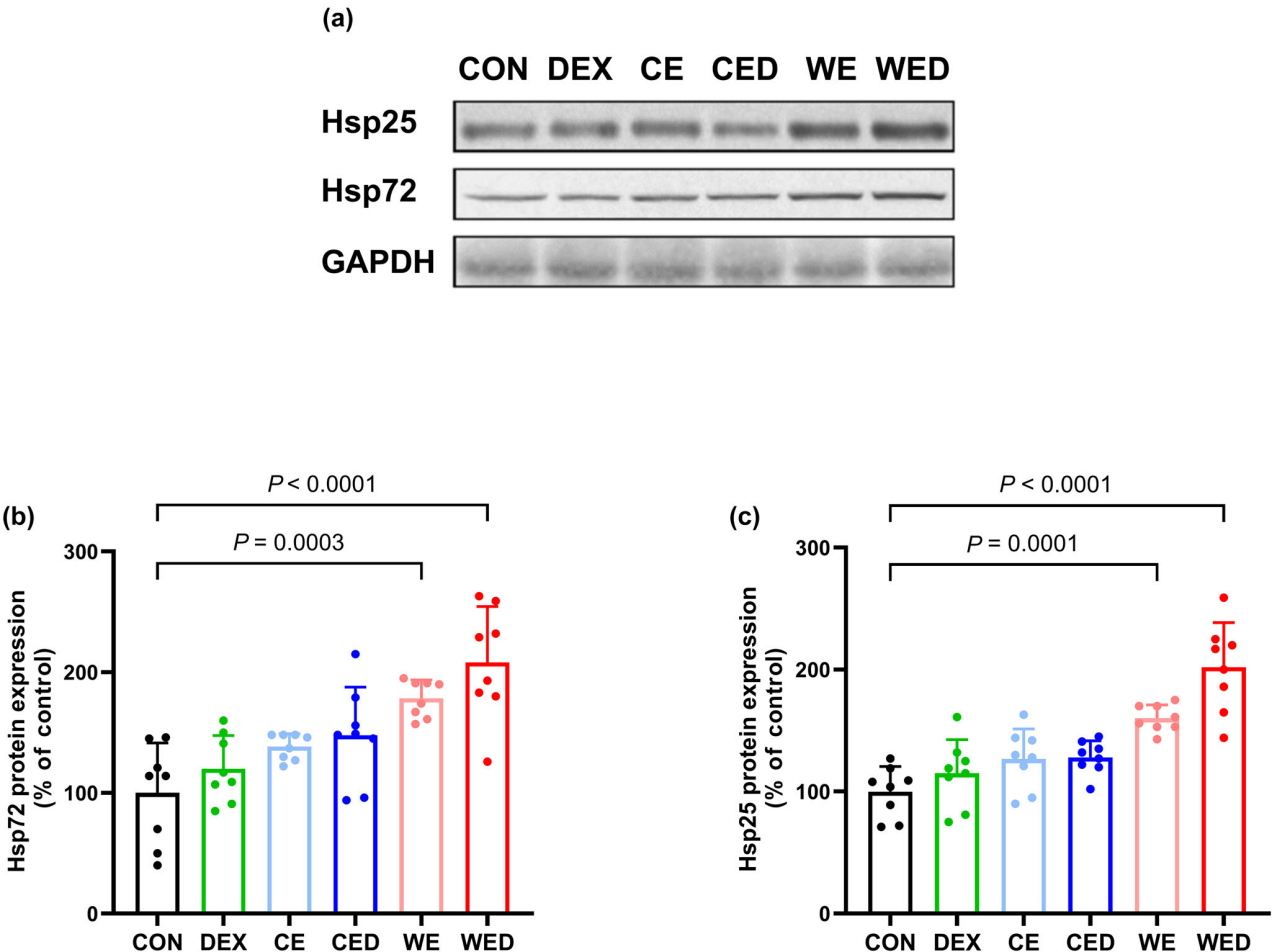
(a) Hsp72 protein expression in plantaris muscle. (b) Hsp25 protein expression in plantaris muscle. (c) The representative western blot image displays Hsp72 and Hsp25 proteins. All protein expression data are presented as mean ± SD (*n* = 8 per group). Differences between groups were analysed using one‐way ANOVA followed by Tukey's *post hoc* test. Abbreviations: CE, exercise in cold environmental conditions; CED, cold exercise with dexamethasone; CON, control; DEX, dexamethasone‐treated; WE, exercise in warm environmental conditions; WED, warm exercise with dexamethasone.

Western blot analyses revealed distinct differences in heat shock protein expression between exercise performed in cold (∼5°C) and warm (25°C) environmental conditions (Figure 6a). As shown in Figure 3, exercise induced a significant increase in rectal temperature in the warm‐environment exercise groups (WE and WED), whereas no comparable elevation was observed in the cold‐environment exercise groups (CE and CED). Consistent with this pattern, Hsp72 protein expression in the plantaris muscle was significantly increased in the WE (vs. CON, *P =* 0.0001) and WED groups (vs. CON, *P* < 0.0001), whereas no changes were observed in the CE and CED groups (*P =* 0.2422) (Figure 6b). Likewise, Hsp25 expression was elevated in the WE group (vs. CON, *P =* 0.0001), with no significant differences in the CE or CED groups (*P =* 0.1961) (Figure 6c).

### 3.3 Effects of exercise in different thermal conditions on Akt–FoxO3a signalling

**FIGURE 7:**
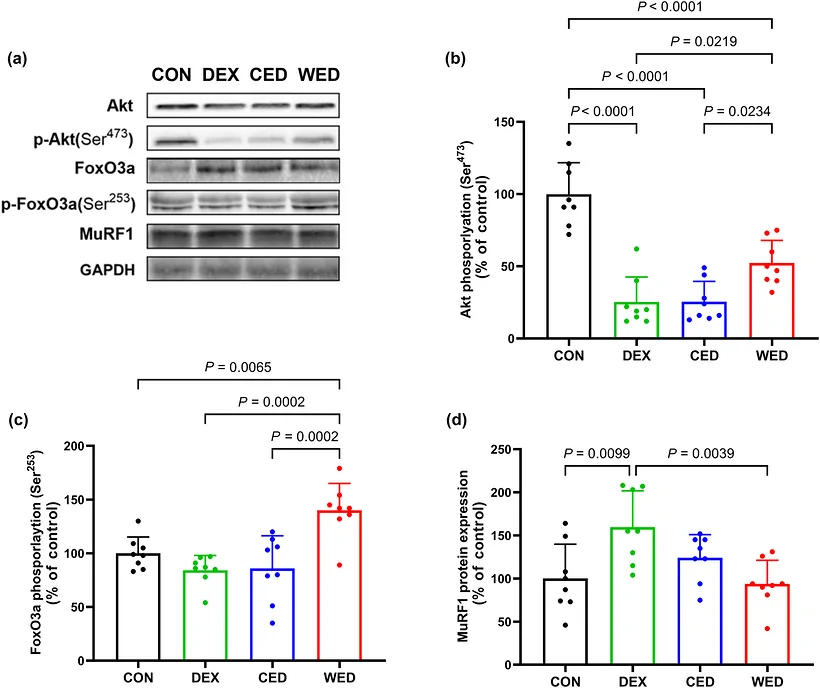
Effects of dexamethasone treatment and exercise at different environmental temperatures on Akt‐FoxO3a signalling and MuRF1 expression in the plantaris muscle. (a) Each protein is displayed on the representative western blot image. (b) Phosphorylation of Akt at Ser473. (c) Phosphorylation of FoxO3a at Ser253. (d) MuRF1 protein expression. All protein expression and phosphorylation data are presented as the mean ± SD (*n* = 8 per group). Differences between groups were analysed using one‐way ANOVA followed by Tukey's *post hoc* test. Abbreviations: CE, exercise in cold environmental conditions; CED, cold exercise with dexamethasone; CON, control; DEX, dexamethasone‐treated; WE, exercise in warm environmental conditions; WED, warm exercise with dexamethasone.

Figure 7 shows the impact of dexamethasone treatment and exercise performed at different environmental temperatures on Akt–FoxO3a signalling and MuRF1 expression in the plantaris muscle. Phosphorylation of Akt at Ser473 was significantly reduced in the dexamethasone‐only group compared with control groups (CON vs. DEX, *P* < 0.0001). A similar reduction was observed in the cold‐environment exercise plus dexamethasone group (CON vs. CED, *P* < 0.0001). In contrast, Akt phosphorylation was significantly higher in the warm‐environment exercise plus dexamethasone group (WED) compared with both the DEX group (*P =* 0.0219) and CED group (*P =* 0.0234); however, it remained lower than control values (*P* < 0.0001).

Consistent with these changes in Akt signalling, dexamethasone treatment was associated with alterations in FoxO3a phosphorylation (Figure 7c). Phosphorylation of FoxO3a at Ser253 was significantly higher in the WED group compared with CON (*P =* 0.0065) and with the DEX and CED groups (*P =* 0.0002 for both), whereas no significant differences were observed between the DEX and CED groups.

MuRF1 protein expression was significantly increased in the DEX group compared with CON (*P =* 0.0099; Figure 7d), consistent with the representative immunoblot (Figure 7a). The CED group showed no attenuation of the DEX‐induced increase and remained comparable to DEX, whereas this increase was attenuated in the WED group (DEX vs. WED, *P =* 0.0039).

Overall, these findings indicate that exercise performed at a warm temperature partly preserves Akt–FoxO3a signalling and attenuates dexamethasone‐induced upregulation of MuRF1. In contrast, exercise in cold conditions does not confer a comparable modulation of this catabolic pathway.

## 4 DISCUSSION

Glucocorticoids are widely used for their potent immunosuppressive properties; however, prolonged exposure is associated with detrimental effects on multiple organ systems, including skeletal muscle (Buchman, 2001; Schakman et al., 2013). Many of the adverse effects of dexamethasone, particularly those affecting muscle tissue, are difficult to reverse and ultimately manifest as skeletal muscle atrophy.

Exercise is known to activate multiple protective mechanisms that counteract glucocorticoid‐induced skeletal muscle atrophy. These responses encompass a broad range of adaptations, including modulation of signalling pathways regulating muscle protein synthesis and degradation, activation of cellular stress responses, and metabolic adjustments (Egan & Sharples, 2023). Among these exercise‐associated responses, elevations in body temperature represent a physiological factor that accompanies exercise and might influence the magnitude of muscle preservation. However, its relative contribution remains incompletely understood. In the present study, we addressed this issue by applying identical exercise protocols in warm and cold environmental conditions, thereby inducing distinct body temperature responses during training and allowing evaluation of the potential modulatory role of temperature‐related mechanisms.

The present findings demonstrate that exercise performed in conditions associated with greater elevations in body temperature was accompanied by a more pronounced attenuation of dexamethasone‐induced skeletal muscle atrophy. Specifically, exercise in the warm environment attenuated reductions in plantaris muscle mass and myofibre cross‐sectional area to a greater extent than exercise performed in cold conditions. Although exercise at ∼5°C also partly mitigated DEX‐induced atrophy, muscle mass and fibre size were not fully restored to control levels, indicating a temperature‐dependent difference in the magnitude of the protective response.

DEX‐induced skeletal muscle atrophy has primarily been attributed to the suppression of muscle protein synthesis pathways, notably the IGF‐1–Akt signalling axis, alongside activation of proteolytic processes (Menconi et al., 2007; Schakman et al., 2013). Consistent with previous reports (Huang et al., 2018; Liu et al., 2016), DEX treatment in the present study resulted in significant reductions in plantaris muscle mass and myofibre CSA. Importantly, these deleterious effects were most effectively attenuated in animals exercising in warm environmental conditions, suggesting that factors associated with elevated body temperature might contribute to the preservation of muscle integrity during glucocorticoid exposure.

Heat stress has been shown to attenuate dexamethasone‐induced myotube diameter loss in C2C12 cells by activating the PI3K–Akt pathway and suppression of FoxO signalling (Tsuchida et al., 2017). Extending these observations to an in vivo setting, the present study demonstrates that exercise‐induced elevations in body temperature are associated with enhanced engagement of anti‐atrophic signalling responses during glucocorticoid treatment, including modulation of Akt–FoxO signalling and induction of heat shock proteins.

One potential mechanism underlying this temperature‐associated protection is the induction of Hsp72, a molecular chaperone known to exert cytoprotective effects in skeletal muscle. Independent of exercise, both transgenic and pharmacological overexpression of Hsp72 has been shown to protect skeletal muscle against various atrophic stimuli. For example, Senf et al. (2008) reported that Hsp72 overexpression prevents immobilization‐induced muscle atrophy by inhibiting FoxO3a transcriptional activity. In the present study, exercise performed in conditions eliciting greater body temperature elevations was accompanied by increased Hsp72 expression, suggesting that Hsp72 induction might represent one mechanism contributing to the enhanced attenuation of catabolic signalling observed in the warm‐exercise group.

Another pathway through which Hsp72 has been proposed to influence muscle mass regulation is the preservation of Akt phosphorylation (Kukreti et al., 2013). Given that DEX is well established to suppress muscle protein synthesis by inhibiting the IGF‐1–Akt signalling pathway (Sandri et al., 2004), we examined whether exercise‐induced differences in body temperature were associated with modulation of Akt signalling. As expected, DEX administration markedly reduced Akt phosphorylation at Ser473. Exercise performed in warm conditions was associated with a partial preservation of Akt phosphorylation relative to both the dexamethasone‐only and cold‐exercise groups. However, levels remained lower than those observed in the control groups. This intermediate maintenance of Akt signalling coincided with increased Hsp72 expression and greater preservation of muscle mass and fibre CSA.

In contrast, Akt phosphorylation in the cold‐exercise group remained comparable to that observed in the dexamethasone‐only group, despite partial attenuation of muscle atrophy. This dissociation between Akt phosphorylation status and morphological outcomes suggests that Akt‐independent mechanisms might contribute to the protective effects of exercise performed in cold conditions.

Beyond these pathways, heat stress also activates heat shock factor‐1 (HSF‐1), which regulates a broad transcriptional network extending beyond classical heat shock proteins. Previous studies have demonstrated interactions between HSF‐1 signalling and metabolic regulators, such as peroxisome proliferator‐activated receptor gamma coactivator 1‐alpha (PGC‐1α), including evidence that HSF‐1 can influence PGC‐1α‐dependent metabolic and mitochondrial programmes (Ma et al., 2015). These observations suggest that heat‐associated responses might influence mitochondrial and metabolic adaptations alongside protein turnover pathways. Accordingly, the protective effects observed in warm‐exercise conditions might involve multiple interacting stress‐responsive mechanisms in addition to pathways related to Hsp72 induction.

With respect to downstream catabolic signalling, phosphorylated FoxO3a levels were not reduced in the DEX group. Nevertheless, MuRF1 protein expression was markedly increased, indicating activation of catabolic transcriptional programmes classically associated with muscle atrophy. Although FoxO‐mediated mechanisms cannot be excluded given their established role in regulating muscle‐specific ubiquitin ligases (Kang et al., 2017), the absence of detectable changes in FoxO3a phosphorylation suggests that additional regulatory pathways might be involved (Fry et al., 2016; Wu et al., 2014).

Previous studies indicate that MuRF1 regulation is not solely dependent on FoxO3a. In disuse‐induced atrophy, MuRF1 promoter activation has been shown to require NF‐κB binding sites, whereas FoxO sites appear not to be essential for this response (Wu et al., 2014). In addition, glucocorticoid‐induced MuRF1 upregulation has been linked to increased expression of NF‐κB‐inducing kinase (NIK) in skeletal muscle (Fry et al., 2016). Together, these findings support the involvement of FoxO‐independent mechanisms in the regulation of MuRF1 in catabolic conditions.

In the warm‐exercise dexamethasone group (WED), FoxO3a phosphorylation was elevated, yet MuRF1 levels remained at control values, coinciding with a pronounced induction of Hsp25. Previous work has demonstrated that Hsp25/27 can inhibit NF‐κB‐mediated MuRF1 transcription without affecting FoxO activity (Dodd et al., 2009), raising the possibility that stress‐responsive pathways contributed, alongside FoxO3a phosphorylation, to restrain MuRF1 upregulation in WED. Although NF‐κB or NIK activity was not assessed directly in the present study, these observations suggest that heat‐induced Hsp25 might have modulated catabolic signalling and partly attenuated the MuRF1 response to glucocorticoid exposure.

Exercise represents a multifaceted physiological stimulus that activates multiple, partly overlapping protective pathways. In addition to Akt signalling, exercise has been shown to enhance antioxidant defence systems (Powers et al., 2020), optimize intracellular Ca^2+^ handling, and improve mitochondrial bioenergetics and quality control processes (Memme et al., 2021). Although these pathways were not assessed directly in the present study and therefore represent a limitation, they might act in concert with temperature‐sensitive responses, including Hsp72 induction, to contribute to the overall protective effects of exercise observed in both thermal conditions.

Studies in rats have demonstrated that identical exercise performed in warm (25°C), but not cold (4°C), environmental conditions increases body temperature, augments skeletal muscle Hsp72 expression and activates Akt signalling (Ogura et al., 2008; Tsuzuki et al., 2018). Although the exercise protocols applied in the cold‐ and warm‐exercise groups were matched for workload and duration, the physiological responses to the same absolute exercise might differ in cold and warm environmental conditions. Accordingly, the higher rectal temperatures observed in the warm‐exercise group in the present study are consistent with these previous findings (Ogura et al., 2008; Tsuzuki et al., 2018). From a physiological perspective, muscles enriched in fast‐twitch glycolytic fibres, such as the plantaris, are particularly susceptible to dexamethasone‐induced atrophy (Livingstone et al., 1981). Therefore, the greater elevation in body temperature achieved during warm‐environment exercise might have enhanced activation of protective signalling pathways, thereby providing greater attenuation of glucocorticoid‐induced muscle loss.

Collectively, these data indicate that exercise‐induced elevations in body temperature are associated with enhanced engagement of protective signalling responses during glucocorticoid exposure, including modulation of Hsp72 and Akt‐associated pathways, while not excluding the contribution of temperature‐independent mechanisms.

### 4.1 Limitations and future directions

Several limitations of the present study should be acknowledged. First, although increased Hsp72 expression was observed in association with greater attenuation of catabolic signalling in the warm‐exercise group, no experimental approach was taken to inhibit Hsp72 induction selectively during exercise. Consequently, a direct causal link between Hsp72 upregulation and muscle preservation cannot be established. Future studies incorporating genetic or pharmacological strategies will be required to test the contribution of Hsp72 directly.

Second, signalling analyses were conducted at a single postintervention time point. Given the dynamic regulation of Akt and FoxO3a phosphorylation, transient or earlier signalling events might not have been captured. Time course analyses might therefore provide additional insight into the temporal interplay between exercise, temperature and catabolic signalling pathways.

Additionally, metabolic indices, such as lactate accumulation and muscle glycogen utilization, were not assessed. Given that exercise in warm conditions was associated with greater elevations in body temperature, future studies integrating metabolic measurements might help to clarify the role of metabolic stress in the temperature‐dependent modulation of exercise responses.

## AUTHOR CONTRIBUTIONS

Conception or design of the work: Berkay Ozerklig, Senay Akin, Bulent Okan Yildiz, Scott K. Powers, Ibrahim Turkel, Haydar A. Demirel; acquisition or analysis or interpretation of data for the work: Berkay Ozerklig, Senay Akin, Seda Olgaz Bingol, Haydar A. Demirel; drafting the work or revising it critically for important intellectual content: Berkay Ozerklig, Senay Akin, Scott K. Powers, Seda Olgaz Bingol, Haydar A. Demirel All authors have read and approved the final version of this manuscript and agree to be accountable for all aspects of the work in ensuring that questions related to the accuracy or integrity of any part of the work are appropriately investigated and resolved. All persons designated as authors qualify for authorship, and all those who qualify for authorship are listed.

## CONFLICT OF INTEREST

None declared.

## GENERATIVE AI STATEMENT

The authors confirm that no artificial intelligence tools, including large language models (LLMs), were used in the drafting or revision of this manuscript. All content was conceived, written and approved solely by the authors.

## Data Availability

The datasets generated and/or analysed during the preent study are available from the corresponding author upon reasonable request.

## References

[eph70444-bib-0001] Akin, S. , Naito, H. , Ogura, Y. , Ichinoseki‐Sekine, N. , Kurosaka, M. , Kakigi, R. , & Demirel, H. A. (2017). Short‐term treadmill exercise in a cold environment does not induce adrenal Hsp72 and Hsp25 expression. Journal of Physiological Sciences, 67(3), 407–413. 10.1007/s12576-016-0473-0 PMC10717108 27470130

[eph70444-bib-0002] Bodine, S. C. , Stitt, T. N. , Gonzalez, M. , Kline, W. O. , Stover, G. L. , Bauerlein, R. , Zlotchenko, E. , Scrimgeour, A. , Lawrence, J. C. , Glass, D. J. , & Yancopoulos, G. D. (2001). Akt/mTOR pathway is a crucial regulator of skeletal muscle hypertrophy and can prevent muscle atrophy in vivo. Nature Cell Biology, 3(11), 1014–1019. 11715023 10.1038/ncb1101-1014

[eph70444-bib-0003] Braun, T. P. , & Marks, D. L. (2015). The regulation of muscle mass by endogenous glucocorticoids. Frontiers in Physiology, 6, 12. 25691871 10.3389/fphys.2015.00012 PMC4315033

[eph70444-bib-0004] Brooks, S. V. , & Faulkner, J. A. (1988). Contractile properties of skeletal muscles from young, adult and aged mice. The Journal of Physiology, 404(1), 71–82. 3253447 10.1113/jphysiol.1988.sp017279 PMC1190815

[eph70444-bib-0005] Buchman, A. L (2001). Side effects of corticosteroid therapy. Journal of Clinical Gastroenterology, 33(4), 289–294. 11588541 10.1097/00004836-200110000-00006

[eph70444-bib-0006] Criswell, D. , Powers, S. , Dodd, S. , Lawler, J. , Edwards, W. , Renshler, K. , & Grinton, S. (1993). High intensity training‐induced changes in skeletal muscle antioxidant enzyme activity. Medicine and Science in Sports and Exercise, 25(10), 1135–1140. 8231758

[eph70444-bib-0007] Demirel, H. A. , Powers, S. K. , Zergeroglu, M. A. , Shanely, R. A. , Hamilton, K. , Coombes, J. , & Naito, H. (2001). Short‐term exercise improves myocardial tolerance to in vivo ischemia‐reperfusion in the rat. Journal of Applied Physiology, 91(5), 2205–2212. 11641363 10.1152/jappl.2001.91.5.2205

[eph70444-bib-0008] Dodd, S. L. , Hain, B. , Senf, S. M. , & Judge, A. R. (2009). Hsp27 inhibits IKKbeta‐induced NF‐kappaB activity and skeletal muscle atrophy. Federation of American Societies for Experimental Biology Journal, 23(10), 3415–3423. 19528257 10.1096/fj.08-124602 PMC2747674

[eph70444-bib-0009] Egan, B. , & Sharples, A. P. (2023). Molecular responses to acute exercise and their relevance for adaptations in skeletal muscle to exercise training. Physiological Reviews, 103(3), 2057–2170. 36395350 10.1152/physrev.00054.2021

[eph70444-bib-0010] Falduto, M. T. , Czerwinski, S. M. , & Hickson, R. C. (1990). Glucocorticoid‐induced muscle atrophy prevention by exercise in fast‐twitch fibers. Journal of Applied Physiology, 69(3), 1058–1062. 2246153 10.1152/jappl.1990.69.3.1058

[eph70444-bib-0011] Fappi, A. , Neves, J. D. C. , Sanches, L. N. , Massaroto e Silva, P. V. , Sikusawa, G. Y. , Brandão, T. P. C. , Chadi, G. , & Zanoteli, E. (2019). Skeletal Muscle Response to Deflazacort, Dexamethasone and Methylprednisolone. Cells, 8(5), 406. 31052442 10.3390/cells8050406 PMC6562646

[eph70444-bib-0012] Fennel, Z. J. , Ducharme, J. B. , Berkemeier, Q. N. , Specht, J. W. , McKenna, Z. J. , Simpson, S. E. , Nava, R. C. , Escobar, K. A. , Hafen, P. S. , Deyhle, M. R. , Amorim, F. T. , & Mermier, C. M. (2023). Effect of heat stress on heat shock protein expression and hypertrophy‐related signaling in the skeletal muscle of trained individuals. American Journal of Physiology‐Regulatory, Integrative and Comparative Physiology, 325(6), R735–R749. 37842742 10.1152/ajpregu.00031.2023

[eph70444-bib-0013] Fry, C. S. , Nayeem, S. Z. , Dillon, E. L. , Sarkar, P. S. , Tumurbaatar, B. , Urban, R. J. , Wright, T. J. , Sheffield‐Moore, M. , Tilton, R. G. , & Choudhary, S. (2016). Glucocorticoids increase skeletal muscle NF‐kappaB inducing kinase (NIK): Links to muscle atrophy. Physiological Reports, 4(21), e13014. 27905294 10.14814/phy2.13014 PMC5112493

[eph70444-bib-0014] Hasselgren, P. O. (1999). Glucocorticoids and muscle catabolism. Current Opinion in Clinical Nutrition and Metabolic Care, 2, 201–205. 10456248 10.1097/00075197-199905000-00002

[eph70444-bib-0015] Hood, D. A. , Memme, J. M. , Oliveira, A. N. , & Triolo, M. (2019). Maintenance of skeletal muscle mitochondria in health, exercise, and aging. Annual Review of Physiology, 81(1), 19–41. 10.1146/annurev-physiol-020518-114310 30216742

[eph70444-bib-0016] Huang, Y. , Chen, K. , Ren, Q. , Yi, L. , Zhu, J. , Zhang, Q. , & Mi, M. (2018). Dihydromyricetin attenuates dexamethasone‐induced muscle atrophy by improving mitochondrial function via the PGC‐1alpha pathway. Cellular Physiology and Biochemistry, 49(2), 758–779. 30165349 10.1159/000493040

[eph70444-bib-0017] Kang, S. H. , Lee, H. A. , Kim, M. , Lee, E. , Sohn, U. D. , & Kim, I. (2017). Forkhead box O3 plays a role in skeletal muscle atrophy through expression of E3 ubiquitin ligases MuRF‐1 and atrogin‐1 in Cushing's syndrome. American Journal of Physiology‐Endocrinology and Metabolism, 312(6), E495–E507. 28246104 10.1152/ajpendo.00389.2016

[eph70444-bib-0018] Kukreti, H. , Amuthavalli, K. , Harikumar, A. , Sathiyamoorthy, S. , Feng, P. Z. , Anantharaj, R. , Tan, S. L. , Lokireddy, S. , Bonala, S. , Sriram, S. , McFarlane, C. , Kambadur, R. , & Sharma, M. (2013). Muscle‐specific microRNA1 (miR1) targets heat shock protein 70 (HSP70) during dexamethasone‐mediated atrophy. Journal of Biological Chemistry, 288(9), 6663–6678. 23297411 10.1074/jbc.M112.390369 PMC3585106

[eph70444-bib-0019] Kumar, V. , Atherton, P. , Smith, K. , & Rennie, M. J. (2009). Human muscle protein synthesis and breakdown during and after exercise. Journal of Applied Physiology, 106(6), 2026–2039. 19164770 10.1152/japplphysiol.91481.2008

[eph70444-bib-0020] Liu, J. , Peng, Y. , Wang, X. , Fan, Y. , Qin, C. , Shi, L. , Tang, Y. , Cao, K. , Li, H. , Long, J. , & Liu, J. (2016). Mitochondrial dysfunction launches dexamethasone‐induced skeletal muscle atrophy via AMPK/FOXO3 signaling. Molecular Pharmaceutics, 13(1), 73–84. 26592738 10.1021/acs.molpharmaceut.5b00516

[eph70444-bib-0021] Livingstone, I. , Johnson, M. A. , & Mastaglia, F. L. (1981). Effects of dexamethasone on fibre subtypes in rat muscle. Neuropathology and Applied Neurobiology, 7(5), 381–398. 6457999 10.1111/j.1365-2990.1981.tb00240.x

[eph70444-bib-0022] Ma, K. , Mallidis, C. , Bhasin, S. , Mahabadi, V. , Artaza, J. , Gonzalez‐Cadavid, N. , Arias, J. , & Salehian, B. (2003). Glucocorticoid‐induced skeletal muscle atrophy is associated with upregulation of myostatin gene expression. American Journal of Physiology‐Endocrinology and Metabolism, 285(2), E363–E371. 12721153 10.1152/ajpendo.00487.2002

[eph70444-bib-0023] Ma, X. , Xu, L. , Alberobello, A. T. , Gavrilova, O. , Bagattin, A. , Skarulis, M. , Liu, J. , Finkel, T. , & Mueller, E. (2015). Celastrol protects against obesity and metabolic dysfunction through activation of a HSF1‐PGC1alpha transcriptional axis. Cell Metabolism, 22(4), 695–708. 26344102 10.1016/j.cmet.2015.08.005

[eph70444-bib-0024] Macedo, A. G. , Krug, A. L. , Herrera, N. A. , Zago, A. S. , Rush, J. W. , & Amaral, S. L. (2014). Low‐intensity resistance training attenuates dexamethasone‐induced atrophy in the flexor hallucis longus muscle. Journal of Steroid Biochemistry and Molecular Biology, 143, 357–364. 24861267 10.1016/j.jsbmb.2014.05.010

[eph70444-bib-0025] Memme, J. M. , Erlich, A. T. , Phukan, G. , & Hood, D. A. (2021). Exercise and mitochondrial health. The Journal of Physiology, 599(3), 803–817. 31674658 10.1113/JP278853

[eph70444-bib-0026] Menconi, M. , Fareed, M. , O'Neal, P. , Poylin, V. , Wei, W. , & Hasselgren, P.‐O. (2007). Role of glucocorticoids in the molecular regulation of muscle wasting. Critical Care Medicine, 35, (Suppl), S602–S608. 17713416 10.1097/01.CCM.0000279194.11328.77

[eph70444-bib-0027] Morimoto, Y. , Kondo, Y. , Kataoka, H. , Honda, Y. , Kozu, R. , Sakamoto, J. , Nakano, J. , Origuchi, T. , Yoshimura, T. , & Okita, M. (2015). Heat treatment inhibits skeletal muscle atrophy of glucocorticoid‐induced myopathy in rats. Physiological Research, 64, 897–905. 26047372 10.33549/physiolres.932942

[eph70444-bib-0028] Naito, H. , Powers, S. K. , Demirel, H. A. , Sugiura, T. , Dodd, S. L. , & Aoki, J. (2000). Heat stress attenuates skeletal muscle atrophy in hindlimb‐unweighted rats. Journal of Applied Physiology, 88(1), 359–363. 10642402 10.1152/jappl.2000.88.1.359

[eph70444-bib-0029] Noble, E. G. , Milne, K. J. , & Melling, C. W. (2008). Heat shock proteins and exercise: A primer. Applied Physiology, Nutrition, and Metabolism, 33(5), 1050–1065. 10.1139/H08-069 18923583

[eph70444-bib-0030] Ogura, Y. , Naito, H. , Akin, S. , Ichinoseki‐Sekine, N. , Kurosaka, M. , Kakigi, R. , Sugiura, T. , Powers, S. K. , Katamoto, S. , & Demirel, H. A. (2008). Elevation of body temperature is an essential factor for exercise‐increased extracellular heat shock protein 72 level in rat plasma. American Journal of Physiology‐Regulatory, Integrative and Comparative Physiology, 294(5), R1600–R1607. 18367652 10.1152/ajpregu.00581.2007

[eph70444-bib-0031] Powers, S. K. , Deminice, R. , Ozdemir, M. , Yoshihara, T. , Bomkamp, M. P. , & Hyatt, H. (2020). Exercise‐induced oxidative stress: Friend or foe? Journal of Sport and Health Science, 9(5), 415–425. 32380253 10.1016/j.jshs.2020.04.001 PMC7498668

[eph70444-bib-0032] Sandri, M. , Sandri, C. , Gilbert, A. , Skurk, C. , Calabria, E. , Picard, A. , Walsh, K. , Schiaffino, S. , Lecker, S. H. , & Goldberg, A. L. (2004). Foxo transcription factors induce the atrophy‐related ubiquitin ligase atrogin‐1 and cause skeletal muscle atrophy. Cell, 117(3), 399–412. 15109499 10.1016/s0092-8674(04)00400-3 PMC3619734

[eph70444-bib-0033] Schakman, O. , Kalista, S. , Barbé, C. , Loumaye, A. , & Thissen, J. P. (2013). Glucocorticoid‐induced skeletal muscle atrophy. International Journal of Biochemistry & Cell Biology, 45(10), 2163–2172. 23806868 10.1016/j.biocel.2013.05.036

[eph70444-bib-0034] Schiaffino, S. , & Mammucari, C. (2011). Regulation of skeletal muscle growth by the IGF1‐Akt/PKB pathway: Insights from genetic models. Skeletal Muscle, 1(1), 4. 21798082 10.1186/2044-5040-1-4 PMC3143906

[eph70444-bib-0035] Senf, S. M. , Dodd, S. L. , & Judge, A. R. (2010). FOXO signaling is required for disuse muscle atrophy and is directly regulated by Hsp70. American Journal of Physiology‐Cell Physiology, 298(1), C38–C45. 19864323 10.1152/ajpcell.00315.2009 PMC2806148

[eph70444-bib-0036] Senf, S. M. , Dodd, S. L. , McClung, J. M. , & Judge, A. R. (2008). Hsp70 overexpression inhibits NF‐kappaB and Foxo3a transcriptional activities and prevents skeletal muscle atrophy. The Federation of American Societies for Experimental Biology Journal, 22(11), 3836–3845. 18644837 10.1096/fj.08-110163 PMC6137947

[eph70444-bib-0037] Shalgi, R. , Hurt, J. A. , Krykbaeva, I. , Taipale, M. , Lindquist, S. , & Burge, C. B. (2013). Widespread regulation of translation by elongation pausing in heat shock. Molecular Cell, 49(3), 439–452. 23290915 10.1016/j.molcel.2012.11.028 PMC3570722

[eph70444-bib-0038] Smuder, A. J. , Morton, A. B. , Hall, S. E. , Wiggs, M. P. , Ahn, B. , Wawrzyniak, N. R. , Sollanek, K. J. , Min, K. , Kwon, O. S. , Nelson, W. B. , & Powers, S. K. (2019). Effects of exercise preconditioning and HSP72 on diaphragm muscle function during mechanical ventilation. Journal of Cachexia, Sarcopenia and Muscle, 10(4), 767–781. 30972953 10.1002/jcsm.12427 PMC6711411

[eph70444-bib-0039] Tsuchida, W. , Iwata, M. , Akimoto, T. , Matsuo, S. , Asai, Y. , & Suzuki, S. (2017). Heat stress modulates both anabolic and catabolic signaling pathways preventing dexamethasone‐induced muscle atrophy in vitro. Journal of Cellular Physiology, 232(3), 650–664. 27649272 10.1002/jcp.25609 PMC5132157

[eph70444-bib-0040] Tsuzuki, T. , Yoshihara, T. , Ichinoseki‐Sekine, N. , Kakigi, R. , Takamine, Y. , Kobayashi, H. , & Naito, H. (2018). Body temperature elevation during exercise is essential for activating the Akt signaling pathway in the skeletal muscle of type 2 diabetic rats. PLOS ONE, 13(10), e0205456. 30304029 10.1371/journal.pone.0205456 PMC6179285

[eph70444-bib-0041] Wu, C. L. , Cornwell, E. W. , Jackman, R. W. , & Kandarian, S. C. (2014). NF‐kappaB but not FoxO sites in the MuRF1 promoter are required for transcriptional activation in disuse muscle atrophy. American Journal of Physiology‐Cell Physiology, 306(8), C762–C767. 24553183 10.1152/ajpcell.00361.2013 PMC3989716

